# Brain endothelial CXCL12 attracts protective natural killer cells during ischemic stroke

**DOI:** 10.1186/s12974-023-02689-x

**Published:** 2023-01-11

**Authors:** Shuaiwei Wang, Lauriane de Fabritus, Praveen Ashok Kumar, Yves Werner, Minglu Ma, Dan Li, Carole Siret, Milesa Simic, Bin Li, Yann M. Kerdiles, Lei Hou, Ralf Stumm, Serge A. van de Pavert

**Affiliations:** 1 Aix-Marseille Univ, CNRS, INSERM, Centre d’Immunologie de Marseille-Luminy (CIML), Marseille, France; 2 Institute of Pharmacology and Toxicology, Jena University Hospital, Jena, Germany; 3 Center for Immune-Related Diseases at Shanghai Institute of Immunology, Department of Respiratory and Critical Care Medicine of Ruijin Hospital, Shanghai Jiao Tong University School of Medicine, Shanghai, China; 4 Institute of Cardiovascular Diseases, Tongren Hospital, Shanghai Jiao Tong University School of Medicine, Shanghai, China

**Keywords:** NK cells, ILC1, Photothrombotic stroke, CNS, Cdh5, CXCR4, Blood–brain barrier, Whole mount imaging, Flow cytometry, Beam-walk sensorimotor test

## Abstract

**Background:**

The innate lymphoid cell (ILC) family consists of NK cells, ILC type 1, 2, 3 and lymphoid tissue inducer cells. They have been shown to play important roles in homeostasis and immune responses and are generally considered tissue resident. Not much is known about the presence of ILC members within the central nervous system and whether they are tissue resident in this organ too. Therefore, we studied the presence of all ILC members within the central nervous system and after ischemic brain insult.

**Methods:**

We used the photothrombotic ischemic lesion method to induce ischemic lesions within the mouse brain. Using whole-mount immunofluorescence imaging, we established that the ILCs were present at the rim of the lesion. We quantified the increase of all ILC members at different time-points after the ischemic lesion induction by flow cytometry. Their migration route via chemokine CXCL12 was studied by using different genetic mouse models, in which we induced deletion of *Cxcl12* within the blood–brain barrier endothelium, or its receptor, *Cxcr4*, in the ILCs. The functional role of the ILCs was subsequently established using the beam-walk sensorimotor test.

**Results:**

Here, we report that ILCs are not resident within the mouse brain parenchyma during steady-state conditions, but are attracted towards the ischemic stroke. Specifically, we identify NK cells, ILC1s, ILC2s and ILC3s within the lesion, the highest influx being observed for NK cells and ILC1s. We further show that CXCL12 expressed at the blood–brain barrier is essential for NK cells and NKp46^+^ ILC3s to migrate toward the lesion. Complementary, *Cxcr4*-deficiency in NK cells prevents NK cells from entering the infarct area. Lack of NK cell migration results in a higher neurological deficit in the beam-walk sensorimotor test.

**Conclusions:**

This study establishes the lack of ILCs in the mouse central nervous system at steady-state and their migration towards an ischemic brain lesion. Our data show a role for blood–brain barrier-derived CXCL12 in attracting protective NK cells to ischemic brain lesions and identifies a new CXCL12/CXCR4-mediated component of the innate immune response to stroke.

**Supplementary Information:**

The online version contains supplementary material available at 10.1186/s12974-023-02689-x.

## Introduction

Ischemic stroke triggers prominent innate immune responses which play multiphasic roles in the pathogenesis of ischemic brain injury. Initially, microglia are rapidly activated after stroke and their activation persists into the later phase, resulting in dual effects for the injury [1]. Subsequently, leukocytes including neutrophils, monocytes and dendritic cells [1–4] are attracted to the lesioned tissue. While neutrophil recruitment is thought to be deleterious, brain infiltration by anti-inflammatory monocytes has been connected to improved outcomes after experimental stroke. A relative new immune cell population is the Innate Lymphoid Cell (ILC) family [5, 6]. ILC consists of 3 major family members, the ILC1, 2 and 3. NK cells are considered part of the ILC1 family, together with the ILC1 and intermediate-ILC1 (intILC1). ILC2s are part of the ILC type 2, while NKp46^+^ and NKp46^–^ ILC3s and LTi cells are part of the type 3 ILC. NK cells have been described to be involved in stroke [7]; however, if any other ILC member is present during stroke is unknown. Since ILCs are critical for the maintenance of homeostasis and tissue remodeling as well as tissue repair under certain circumstances, they can either be beneficial or detrimental to the outcome of the experimental stroke.

Recently, NK cells have been reported in neurological diseases, including experimental autoimmune encephalomyelitis (EAE), Alzheimer and amyotrophic lateral sclerosis (ALS) and stroke [7–10]. ILC1s and ILC3s are also involved in EAE [8, 11] and ILC3s were shown to be critical for the Th17 response in EAE [12]. However, how ILCs are recruited toward ischemic brain remains largely unknown. The expression of the chemokine CXCL12 and its receptor CXCR4 within the brain increases after stroke, which plays a critical role in the migration of monocytes to brain parenchyma [4]. CXCL12 is constitutively expressed at the blood–brain barrier (BBB) by endothelial cells [13] and redistributed in location during EAE in mice or multiple sclerosis in humans [14], however a role for BBB-derived CXCL12 during ischemic stroke was not known. Given that CXCR4 is expressed by ILCs, and treatment with the CXCR4 antagonist AMD3100 affects the cell numbers of NK cells in the ischemic hemisphere [15–17], CXCL12/CXCR4 signaling could directly regulate NK cell and ILC migration to the infarct region. Indeed, CXCR4^+^ NK cells were shown to extravasate from the blood in patients specifically during the remission phase of multiple sclerosis [18], and were also observed in stroke regions in patients [7]. However, if they respond to CXCL12 expressed in the blood–brain barrier after an ischemic lesion is unknown. Even though ILC populations have been reported in central nervous system (CNS) disorders including EAE and Alzheimer, there is an apparent lack in knowledge on the involvement of ILCs in stroke, and if they are involved in tissue repair or a deleterious immune reaction.

In this study, we show that NK cells, ILC1s, ILC2s, NKp46^+^ ILC3s and LTi cells were recruited to stroke brain, the majority being NK cells and ILC1s. Using Cdh5^CreERT2^;Cxcl12^flox^ mice, in which *Cxcl12* was specifically deleted in BBB endothelial cells, plus a mouse model with *Cxcr4*-deficient NKp46^+^ cells, we demonstrated that BBB CXCL12–CXCR4 axis was essential for the influx of NK cells into the stroke lesion. We further showed that these NK cells protect the brain and improved mouse motor movement after stroke induction.

## Materials and methods

### Mice

RORc^eGFP^ mice were kindly provided by Gérard Eberl (Pasteur Institute, Paris, France) [19]. Ncr1^iCre^ mice were kindly provided by Eric Vivier (CIML, Marseille, France) [20]. Cdh5^CreERT2^ mice were kindly provided by Ralf Adams (Max Planck Institute for Molecular Biomedicine, Münster, Germany) [21]. Cxcr4^flox^, Cxcl12^flox^ and Rosa^tdTomato^ mice were obtained from Jackson Laboratory [22–24]. Except for the 3D imaging analysis, 8- to 14-week-old adult male mice were used. All the mice were housed in the specific pathogen-free (SPF) animal facilities and fed with irradiated standard pellet chow and reverse osmosis water. All experiments were performed according to the French ethics committee regulations.

### Photothrombotic stroke model

Male mice were anesthetized by intraperitoneal injection of ketamine/xylazine and immobilized on a stereotaxic frame. An incision along the midline from the eye level down to the neck was made and the illuminated area on the exposed skull was determined corresponding to the following coordinates: 2.5 mm posterior to bregma and 2.0 mm lateral to midline. A fiber optic connected to KL 1600LED cold light source was placed on the targeting area after 100 μl of Rose Bengal (10 mg/ml) was injected. Illuminating for 15 min activated the Rose Bengal. After the skin was stitched, animals were put on a heating pad and returned to their home cages until they were fully awake.

### Beam-walk sensorimotor test

Mice were trained to walk across a 60-cm-long beam of 0.8 cm diameter, for a minimum of 4 days prior to surgery. For each trial, the mouse was placed at the start and allowed to cross the beam and was given 45 s to reach the goal cage between trials. Each mouse was given five consecutive trials on each test day. The total number of forelimb and hindlimb faults was scored by video recordings. For in vivo depletion of NK cells, mice were injected 100 μg of InVivoPlus anti-mouse Nk1.1 (Clone PK136, Bio X Cell) or InVivoPlus mouse IgG2a (Clone C1.18.4, Bio X Cell) resolved in PBS at P-9 and P-2, respectively.

### Administration of 4-hydroxytamoxifen

4-Hydroxytamoxifen (4OHT—Sigma T176) was prepared in 667 μl of ethanol and 6 ml of peanut oil. 160 μl of 4OHT was administered intraperitoneally on 3 consecutive days [25]. Mice underwent 3 weeks of 4OHT before stroke induction.

### Flow cytometry

Brains were digested in HBSS containing 0.075 mg/ml liberase TM and 0.2 mg/ml DNase l at 37 ℃ for 60 min. After being harvested and washed by HBSS containing 2% FBS, the brain cells suspended in 30% Percoll were centrifuged at 700 G for 15 min, resuspended in FACS buffer (HBSS containing 2% FBS). The skull was placed in a petri dish containing cold PBS, the edge of which was scored 360° with fine forceps. Then the dura maters were carefully peeled off from the interior side of the skull cap. Dura maters isolated from skull as well as deep cervical, mandibular, axillary and mesenteric lymph nodes were digested in HBSS containing 0.075 mg/ml liberase^TM^ and 0.2 mg/ml DNase l at 37 ℃ for 20 min. Incubating collected blood with 1 X RBC lysis buffer (Invitrogen, 2139995) for 30 s on ice, then stopping the reaction by HBSS containing 2% FBS. Cell suspensions were centrifuged at 300 G for 7 min and then resuspended in FACS buffer. In the FACS experiments for Fig. 2, Additional file 1: Figs. S1 and S2, cells were blocked in FACS buffer containing 15% normal mouse serum for 15 min and subsequently stained for CD45 (BD biosciences, 564279), CD3 (BD biosciences, 562286), CD8 (BD biosciences, 562283), CD19 (BD biosciences, 562291), Ly6G (BD biosciences, 562700), F4/80 (BD biosciences, 565613), NKp46 (BD biosciences, 562850), CD127 (Ebioscience, 50-1271-80), ST2 (Ebioscience, 12-9333-80), KLRG1 (BD biosciences, 563595), CD4 (BD biosciences, 561099) diluted in FACS buffer for 30 min on ice. The live and dead cells were distinguished using LIVE/DEAD Fixable Blue Dead Cell Stain Kit (Life technologies, L23105). Cells were subsequently washed and resuspended in FACS buffer. Cells were blocked in FACS buffer containing 15% normal mouse serum for 15 min and subsequently stained for CD45, CD3e, CD8a, CD19, Ly6G, TCRβ, F4/80, NKp46, NK1.1, CD49a, CD49b, CD127 (Ebioscience, 50-1271-80), ST2, KLRG1 diluted in FACS buffer for 30 min on ice. Cells were washed with FACS buffer and resuspended in FACS buffer containing Sytox Blue. Samples were acquired on the BD LSR Fortessa-X20 cytometer and data were analyzed using FlowJo (version 10, LLC) software.

### Immunofluorescence

The mice were perfused with PBS containing 5U/ml of Heparin followed by 4% paraformaldehyde (PFA). The brains were extracted and fixed by 4% PFA overnight at 4 ℃. 50-μm Vibratome sections were cut and blocked in EBT buffer (EBSS with 1% BSA and 2% Triton-X100) containing 10% serum for 1 h at room temperature. Subsequently, sections were incubated with primary antibodies diluted in EBT buffer containing 3% serum for 24 h at 4 ℃. After washing 3 times with PBS containing 0.2% Triton-X100, the sections were incubated with fluorochrome-coupled secondary antibodies diluted in EBT buffer containing 3% serum for 12 h at 4 ℃. After washing 3 times with PBS, the sections were cleared in Histodenz medium (4 g Histodenz (Sigma D2158) in 3 ml of 0.02 M phosphate buffer) for 24 h at room temperature and then mounted in Histodenz medium. Confocal images were acquired on a Zeiss-LSM880 confocal microscope and processed by ImageJ.

### Nissl staining

Vibratome sections were cut and mounted on positive charged plus slides (ThermoFisher scientific, SuperFrost Plus). The slides were placed in 1:1 alcohol/chloroform overnight and then rehydrated through 100% and 95% alcohol. Then the slides were placed into 0.1% cresyl violet for 10 min and rinsed quickly in distilled water. The sections were cleared with xylene for 10 min after being differentiated in 95% alcohol for 5 min and dehydrated in 100% alcohol for 10 min. For quantification of the infarct volumes, the infarct area was measured by using ImageJ and the total volume was calculated as the sum of infarct areas multiplied by the section interval.

### Whole mount immunofluorescence

The *RORc*^*eGFP*^ mice were perfused with PBS containing 5U/ml of heparin followed by 0.4% paraformaldehyde (PFA). The brains were harvested and fixed by 0.4% PFA overnight at 4 ℃ and subsequently placed in permeabilization solution for 3 days. Subsequently, they were incubated in blocking medium PBS-MT (1% skim milk, 0.4% Triton X-100, 5% NDS and 5% NGS) for 3 days and subsequently incubated with anti-GFP (AVES, GFP-1020), anti-CD3 (BD biosciences, 563024), anti-KLRG1 (BD biosciences, 563595) and anti-NKp46 (BD biosciences, 562850) in PBS-MT with 3% NDS plus NGS for 10 days. After washing with PBS containing 0.2% Triton X-100, brains were incubated with donkey anti-chicken Cy3 (Jackson Immunoresearch, 703-166-155), goat anti-Armenian hamster 647 (Jackson Immunoresearch, 127-605-160), goat anti-Syrian hamster 488 and donkey anti-rat 790 (Jackson Immunoresearch, 712-655-153) in PBS-MT with 3% NDS as well as NGS for 10 days. Brains were washed with PBS and dehydrated by methanol series (20%, 40%, 60%, 80% 1 h each and 100% 2 h). Subsequently, the brains were placed in 66% dicholoromethane (Sigma, 270997), 33% methanol overnight, and afterwards in 100% dicholoromethane for 15 min. The brains were cleared in BABB (2:1 benzyl alcohol (Honeywell, 108006)/benzyl benzoate (Acros organics, 105862500)) overnight. Brains were acquired using the light sheet Ultramicroscope version II (LaVision BioTec). 3D imaging analysis were performed using Imaris software (Version 9.1.0, Bitplane).

### Quantitative PCR

Total RNA was prepared by TRIzol reagent (Sigma). cDNA synthesis was carried out using RevertAid RT Kit (ThermoFisher scientific). The transcripts were amplified on the 7500 Fast Real-Time PCR System. The amplification of *Cxcr4* and the corresponding *Gapdh* was detected by Taqman probes (Cxcr4-Mm01292123_m1, Gapdh-Mm99999915_g1; ThermoFisher scientific), combining with Taqman Fast Advanced Master Mix (ThermoFisher scientific). The amplification of *Cxcl12* and the corresponding *Gapdh* was detected by the following primers: *Cxcl12*_forward 5’-tcaagcatctgaaaatcctcaaca-3’, *Cxcl12*_reverse 5’-ttcgggtcaatgcacacttgt-3’, *Gapdh*_forward 5’-tgtgtccgtcgtggatctga-3’, *Gapdh*_reverse 5’-cctgcttcaccaccttcttga-3’, combining with TB Green Premix Ex Taq (Takara). Relative expression was calculated by ∆∆C_T_ method.

### Statistical analysis

Graphs in all figures were analyzed using GraphPad Prism (v.7.03) software. Statistical significance of differences between groups in Figs. 2B, 3E, 4E, 5C and E were analyzed by two-way ANOVA, and in Figs. 4F and 5D were compared using *t*-test. All the cartoons were generated using BioRender.

### Staining panels for FACS

Staining panel I for FACS in Fig. 2, Additional file 1: Figs. S1 and S2.

**Table:** 

CD45	BV395	BD biosciences, 564279
CD3	PE-CF594	BD biosciences, 562286
CD8	PE-CF594	BD biosciences, 562283
CD19	PE-CF594	BD biosciences, 562291
Ly6G	PE-CF594	BD biosciences, 562700
F4/80	PE-CF594	BD biosciences, 565613
NKp46	BV421	BD biosciences, 562850
CD127	eFluor 660	Ebioscience, 50-1271-80
ST2	PE	Ebioscience, 12-9333-80
KLRG1	PerCP-Cy5.5	BD biosciences, 563595
CD4	PE-Cy7	BD biosciences, 561099
RORγt	RORγt-eGFP	Sparwasser et al., 2004
ReporterLive/Dead	Fixable Blue Dead Cell Stain Kit	Life technologies, L23105

Staining panel II for FACS in Figs. 3, 4, 5, Additional file 1: Figs.S4, S6 and S7.

**Table:** 

CD45	BV395	BD biosciences, 564279
CD3	BV510	BD biosciences, 563024
CD8	V500	BD biosciences, 560776
CD19	BV510	BD biosciences, 562956
Ly6G	BV510	Biolegend, BLE127633
F4/80	BV510	BD biosciences, 743280
TCRβ	BV510	BD biosciences, 563221
NKp46	BV421	BD biosciences, 562850
NK1.1	Alexa Fluor 700	Ebioscience, 56-5971-82
CD49a	BV711	BD biosciences, 564863
CD49b	PE-Cy7	Miltenyi, 130-116-370
CD127	eFluor 660	Ebioscience, 50-1271-80
ST2	PE	Ebioscience, 12-9333-80
KLRG1	PerCP-Cy5.5	BD biosciences, 563595
Live/Dead	Sytox Blue	Life technologies, S34857

## Results

### ILCs are present within the stroke lesion

**Fig. 1:**
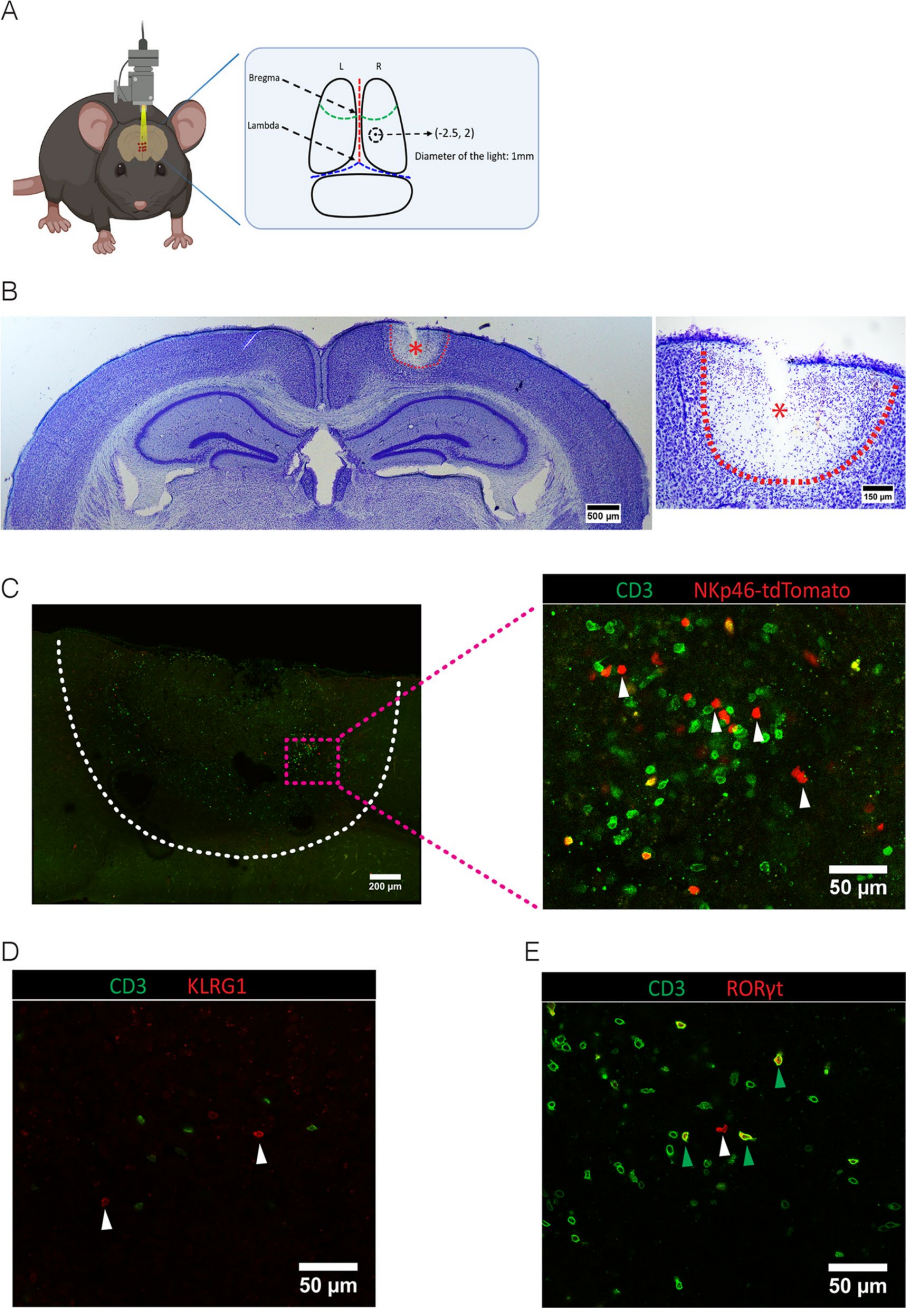
Presence of ILCs in the ischemic stroke lesion. **A** Coordinates of the lesion on the brain of photothromobotic (PT) mouse model at 2.5 mm posterior to bregma and 2.0 mm lateral to midline with 1 mm diameter. **B** Nissl staining on the vibratome section to indicate the lesion in the stroke brain at P2. Data represent *n* = 4 mice. **C** Immunofluorescence on the vibratome sections of stroke brain at P10. Since the markers of ILCs, NKp46, KLRG1 and RORγt are also expressed by CD3^+^ non-ILCs, we used CD3 to distinguish NK/ILC1s from NKT cells, ILC2s from regulatory T cells (Tregs) and NKp46^+^ ILC3s from Th17 cells. CD3^−^NKp46^+^ cells (NK, ILC1 or NKP46^+^ ILC3, white arrows). Data represent *n* = 4 mice. **D** CD3^−^KLRG1^+^ cells (ILC2, white arrows). Data represent *n* = 4 mice (**E**) CD3^+^RORγt^+^ cells (Th17, green arrows) and CD3^−^RORγt^+^ cells (ILC3, white arrows). Data represent *n* = 4 mice

To analyze whether ILCs are involved in responses towards ischemic stroke we used the photo-thrombosis model (PT [4]), which is non-invasive and generates highly reproducible infarcts (Fig. 1A). After verifying the presence of the cortical lesion by Nissl staining at post-operative day 2 (P2) (Fig. 1B), we determined the presence of ILCs in and near the lesion by immunofluorescence on sections (Fig. 1C–E) or in the whole lesioned brain (Additional file 2: Videos S1 and Additional file 3: S2). We observed CD3^–^NKp46^+^ cells (Fig. 1C), which include NK, ILC1, and ILC3 cells in the lesion. We also detected CD3^–^KLRG1^+^ cells (Fig. 1D), which correspond to ILC2s. The transcription factor RORγt, which identifies ILC3 and the non-ILC Th17 cells, was visualized by using *RORc*^*eGFP*^- reporter mice [19, 26]. This showed CD3^–^RORγt^+^ and CD3^+^RORγt^+^ cells (Fig. 1E), which most likely correspond to CD3^–^RORγt^+^ ILC3s and CD3^+^CD4^+^RORγt^+^ Th17 cells. Notably, the number of CD3^–^RORγt^+^ ILC3s was considerably smaller than the number of CD3^–^NKp46^+^ cells (Fig. 1C, E), suggesting that more CD3^–^NKp46^+^ NK cells than CD3^–^RORγt^+^ ILC3s were attracted. To determine the precise spatial distribution of ILCs within the complete ischemic brain, we performed whole-mount immunofluorescence at P2 and P10 (Additional file 2: Video S1 and Additional file 3: Video S2). We could show via 3D imaging analysis of the lesioned brains that CD3^–^NKp46^+^ cells, CD3^–^KLRG1^+^ cells, and CD3^–^RORγt^+^ cells were located mainly at the border of the lesion at both these time-points.

Moreover, NKp46^+^ ILCs accumulated at the dorsal region of the lesion, whereas ILC2s and NKp46^−^ ILC3s migrated into the deeper region. It shows that many ILCs involved in ischemic stroke even they were outnumbered by CD3^+^ cells by quantifying the cell numbers (Table 1).

**Table 1:** CD3^+^ and ILC cell numbers in the stroke lesion at P10

Brain	CD3^+^	CD3^−^NKp46^+^	CD3^−^KLRG1^+^	CD3^−^RORγt^+^	Ratios ILCs/CD3^+^
Mouse #1	16,400	3015	1136	1179	0.33
Mouse #2	18,000	2253	1092	1112	0.25

Number of CD3^+^, CD3^−^NKp46^+^, CD3^−^KLRG1^+^ and CD3^−^RORγt^+^ cells located within the brain lesion (P10) quantified by Image analysis software Imaris on whole-mount 3D lesions. The ratio of ILCs (the sum of CD3^−^NKp46^+^, CD3^−^KLRG1^+^ and CD3^−^RORγt^+^) to CD3^+^ cells was calculated

**Fig. 2:**
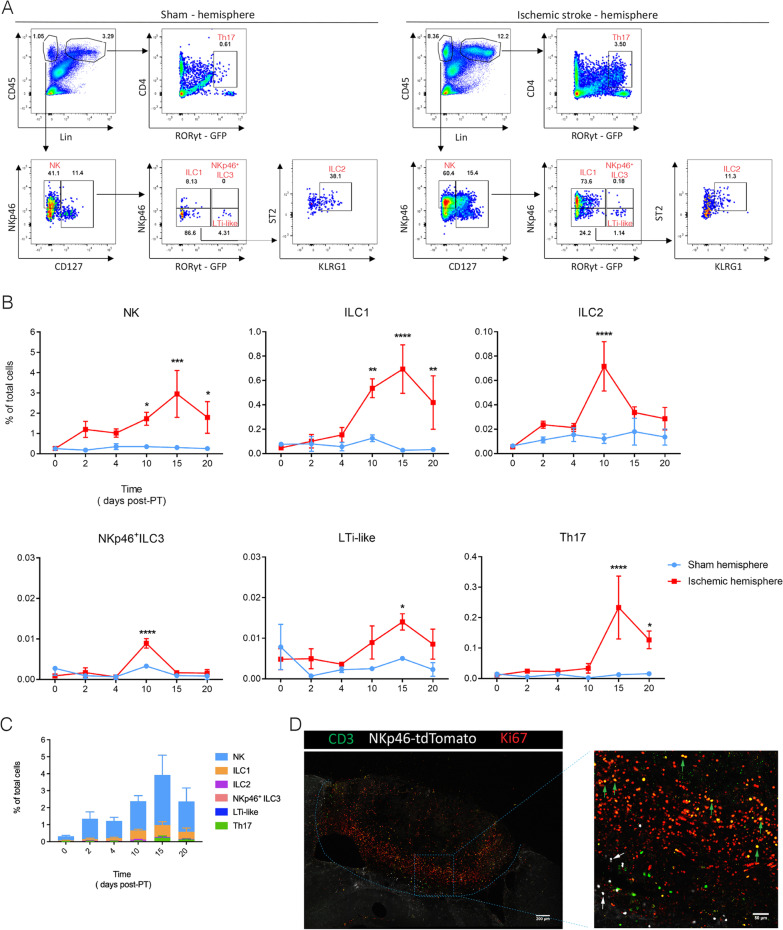
Time-course of ILC migration towards the ischemic stroke lesion. Cytometry analysis of ILCs in the sham (**A**, left) and P15 stroke hemisphere (**A**, right), NK (CD45^+^Lin^−^NKp46^+^CD127^−^), ILC1 (CD45^+^Lin^−^NKp46^+^CD127^+^RORγt^−^), ILC2 (CD45^+^Lin^−^NKp46^−^CD127^+^RORγt^−^ST2^+^KLRG1^+^), NKp46^+^ ILC3 (CD45^+^Lin^−^NKp46^+^CD127^+^RORγt^+^), LTi-like (CD45^+^Lin^−^NKp46^−^CD127^+^RORγt^+^), and Th17 (CD45^+^Lin^+^CD4^+^RORγt^+^). Representative dot plots of ILCs and Th17 cells on P15 are shown with the averages for the gates. Each panel is representative of 3 independent experiments (*n* = 3 mice). (**B**) Quantification of ILC populations and Th17 cells in the hemisphere after PT induction. The percentage of each population in total live cells (named total cells) was calculated. Data represent *n* = 3 mice. NK: P10 *P = 0.0364, P15 ***P = 0.0003, P20 *P = 0.021; ILC1: P10 **P = 0.0052, P15 ****P < 0.0001, P20 **P = 0.008; ILC2: P10 ****P < 0.0001; NKp46^+^ ILC3: ***P = 0.0001; LTi-like: P15 *P = 0.0232; Th17: P10 ****P < 0.0001. **C** Comparison of ILC and Th17 cell numbers in stroke hemisphere at different time points after induction. Data represent *n* = 3 mice. **D** Confocal images showing immunofluorescence for CD3, NKp46 and Ki67 in the PT-induced lesion at day 10. Green arrows point to the proliferating CD3^+^ cells, while white arrows point to the non-proliferating CD3^−^NKp46^+^ cells including NK cells, ILC1s and ILC3s. Data represent *n* = 2 mice

After having established where ILCs were present within the lesioned brain, we set out to quantify all the ILCs within the ischemic brain. In order to do this, we established a flow cytometry staining panel that identified all ILC subsets (gating strategy in Additional file 1: Fig. S1, Fig. 2A). At P0, we observed no or very few ILCs. NK cell numbers increased at P2 (Fig. 2B) and peaked at P15 (Fig. 2B). ILC1 numbers increased at P10 and peaked at P15 (Fig. 2B), but were not detected at P2 and P4 (Fig. 2B). ILC2 numbers sharply increased after P4 and lowered at P15. The number of NKp46^+^ ILC3 cells peaked at P10 while NKp46^–^ LTi-like cells peaked at P15 (Fig. 2B). The presence of Th17 cells was consistent with previous data using the middle cerebral artery occlusion (MCAO) stroke model (Fig. 2B) [27], but their numbers were significantly lower than invading NK cells and ILC1s (Fig. 2C). Summarized, while the immunofluorescence showed the distribution of the ILCs at the rim of the lesion, our flow cytometry analyses allowed the detailed analysis of all ILC subsets in the brain hemisphere undergoing photothrombotic stroke. NK cells and ILC1s constituted the main ILC populations responding to ischemic damage (Fig. 2C). Ki67 immunofluorescence staining showed that some CD3^+^ cells proliferated, but CD3^–^NKp46^+^ cells did not (Fig. 2D). Thus, the progressive increase of NKp46^+^ ILCs is caused by migration towards the lesion rather than in situ proliferation. When the cells do not proliferate and they are not present at steady-state, how do they enter the lesioned brain? It was shown that the dural meninges, the outmost membrane of the cranial meninges, could play a role in migration of T cells in EAE [28]. The dura contains the dural venous sinuses which drain blood and contain shunted lymphatic vessels draining cerebrospinal fluid (CSF) from the brain [29–31]. However, we did not observe a significant increase or decrease in case of migration towards the lesion of ILCs nor Th17 cells in the dura mater after induction of PT (Additional file 1: Figs. S1B, S2A, B), indicating that migration most likely occurs directly towards the lesion.

ILCs have been shown to circulate and migrate towards lymph nodes [32]. Immune cells activated within the brain are drained towards the deep cervical lymph nodes (dcLN) and mandibular lymph nodes (mandiLN) [33]. An increase in ILC numbers within these LNs would indicate an active clearance and/or migration of ILCs toward these structures and a possible involvement of the ILCs in priming the adaptive immune system. We quantified ILCs and Th17 cells within dcLN, mandiLN, mesenteric LN (mLN) and axillary lymph nodes (axiLN) (Additional file 1: Figs. S1C, S2D-G) by taking advantage of flow cytometry.

### Endothelial CXCL12 regulates migration of NK cells and NKp46^+^ ILC3s toward the ischemic brain

**Fig. 3:**
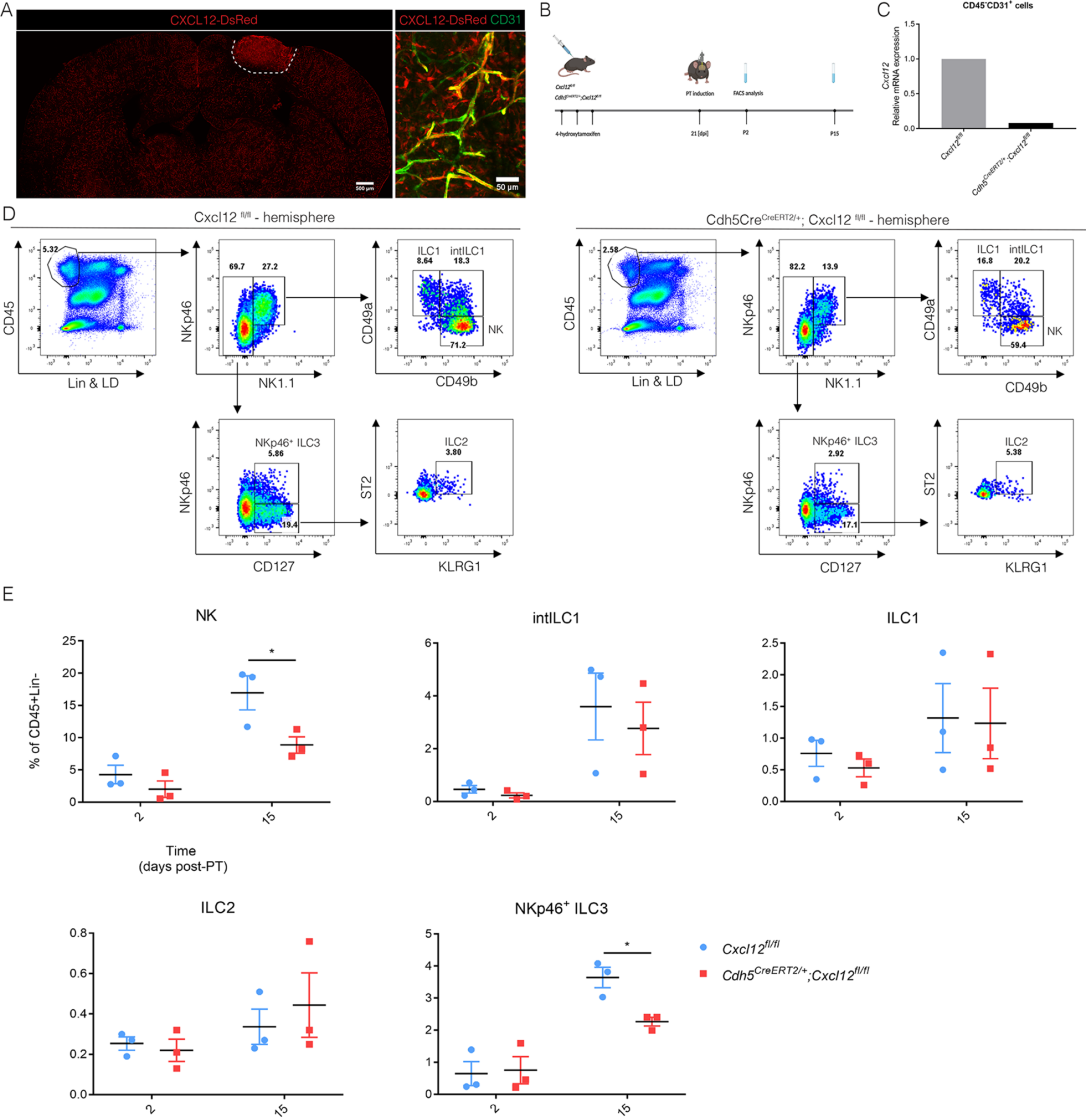
Recruitment of ILCs via blood–brain barrier expressed CXCL12. **A** CXCL12 expression within the lesion at P10, co-localizing with CD31^+^ endothelial cells in *Cxcl12*^*DsRed*^ mouse. Data represent *n* = 2 mice. **B** Cxcl12^fl/fl^ and *Cdh5*^*CreERT2/*+^*;Cxcl12*^*fl/fl*^ mice were injected with 4OHT at day -21 relative to PT induction and analyzed by flow cytometry at day 2 and 15 after PT induction. dpi: days post-injection (**C**) *Cxcl12* mRNA levels in the sorted endothelial cells from WT (Cxcl12^fl/fl^) and KO (*Cxcl12*^*Cdh5–/–*^) mice. RNA was extracted from sorted CD45^+^CD31^+^ cells pooled from 3 mice. **D** Flow cytometry analysis of ILCs in the P15 stroke brain from WT (*Cxcl12*^*fl/fl*^) and KO (*Cxcl12*^*Cdh5–/–*^) mice, NK (CD45^+^Lin^−^NKp46^+^NK1.1^+^CD49a^−^CD49b^+^), intILC1 (CD45^+^Lin^−^NKp46^+^NK1.1^+^CD49a^+^CD49b^+^), ILC1 (CD45^+^Lin^−^NKp46^+^NK1.1^+^CD49a^+^CD49b^−^), ILC2 (CD45^+^Lin^−^NKp46^−^NK1.1^−^CD127^+^ST2^+^KLRG1^+^) and NKp46^+^ ILC3 (CD45^+^Lin^−^NK1.1^−^NKp46^+^CD127^+^). Each panel is representative of 3 independent experiments (*n* = 3 mice). **E** Quantification of ILCs in the stroke hemisphere from Control (*n* = 3 in three independent experiments) (*Cxcl12*^*fl/fl*^) and KO (*Cxcl12*^*Cdh5–/–*^) (*n* = 3 in three independent experiments) mice, at day 2 and 15 after PT induction.. NK *P = 0.0113, NKp46^+^ ILC3 *P = 0.0186

When there are no ILCs present within the brain at steady-state and they do not proliferate within the lesion, how would they migrate towards the lesion? Using in situ hybridization, *Cxcl12* was observed throughout the brain and increased after stroke [4, 13]. Using *Cxcl12-DsRed* reporter mice, we observed CXCL12 expression in CD31^+^ endothelial cells within the stroke lesion at P10 (Fig. 3A). This indicated that CXCL12 is generated by the BBB to attract lymphocytes, as was shown before in brain inflammations [13, 16]. To study whether ILC migration was mediated by BBB-derived CXCL12, we specifically and conditionally deleted endothelial *Cxcl12* using the *Cdh5*^*CreERT2*^*;Cxcl12*^*flox/flox*^ model (from now named *Cxcl12*^*Cdh5–/–*^) since the full *Cxcl12* knock-out is embryonic lethal. 4-Hydroxytamoxifen (4OHT) was injected 3 weeks before PT induction (Fig. 3B) so that the 4OHT was washed out before the experiment [4]. To confirm *Cxcl12* deletion, we sorted CD45^–^CD31^+^ endothelial cells (Additional file 1: Fig. S3A and S3B) and performed a RT-qPCR analysis which confirmed lack of *Cxcl12* within the endothelial cells (Fig. 3C). The presence of NK cells, intILC1s, ILC1s, ILC2s and NKp46^+^ ILC3s in the lesioned brains was determined by flow cytometry (Fig. 3D and Additional file 1: Fig. S4). Compared to the controls, we observed a slight decrease only of NK cells in the *Cxcl12*^*Cdh5–/–*^ stroke brain at P2 and a significant decrease at P15 (Fig. 3E), indicating that the endothelial-produced CXCL12 is directly involved in the attraction of NK cells. NKp46^+^ ILC3s were also decreased within stroke brain at P15 (Fig. 3E). These results demonstrate that BBB-derived CXCL12 mediated migration of NK and NKp46^+^ ILC3s into ischemic stroke brain.

### Migration of NK cells to the stroke lesion depends on CXCR4

**Fig. 4:**
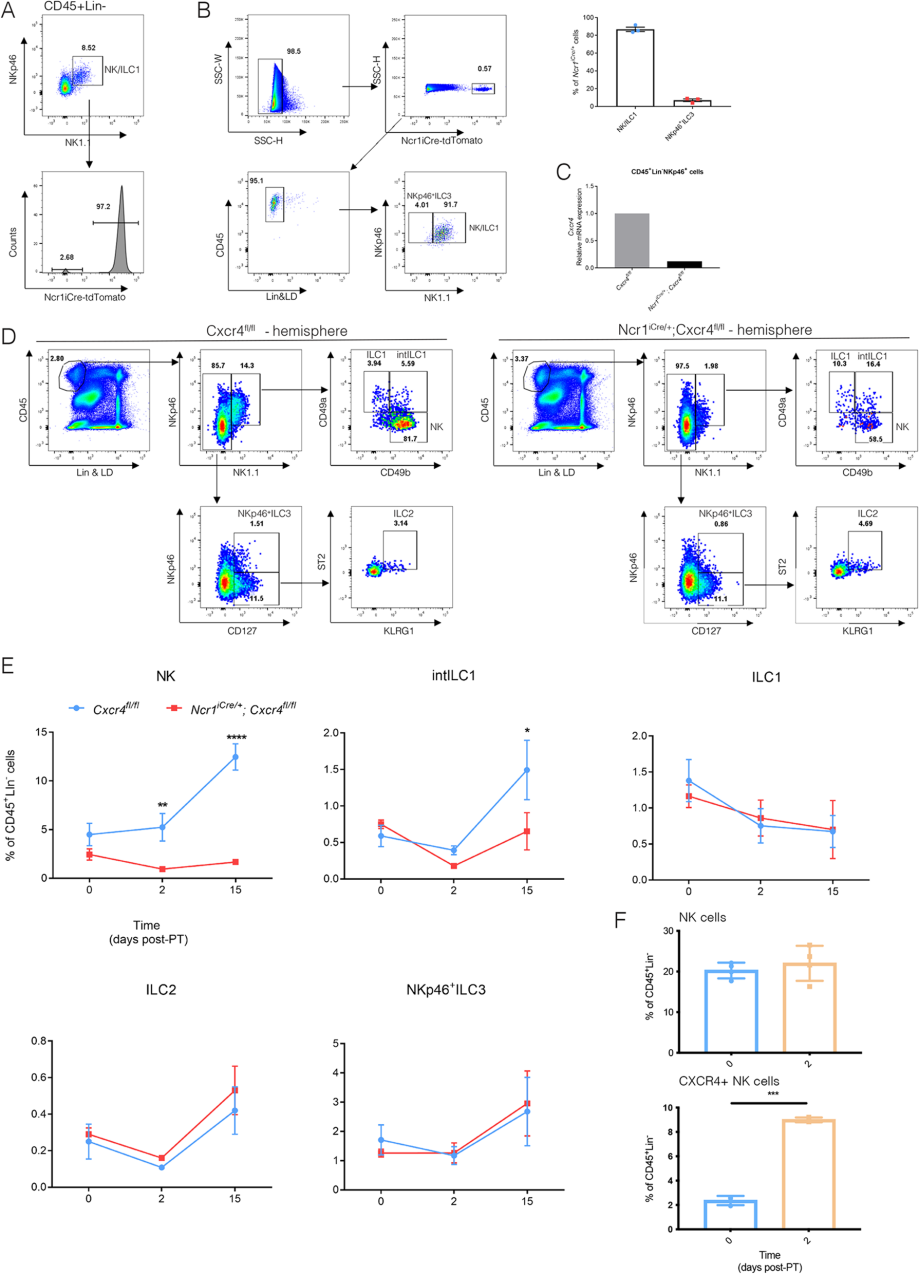
NK cell migration is CXCR4-dependent. **A** Testing the *Ncr*^*iCre*^ efficiency in the NKp46^+^ cells by FACS using the *Rosa*^*tdTomato*^ reporter mice (*n* = 3). **B** Analysis of the NKp46^+^ cell populations in the *Ncr*^*iCre*^; *Rosa*^*tdTomato*^ reporter mice (*n* = 3). Representative dot plots at P15 are shown. **C** *Cxcr4* mRNA levels in sorted NK cells from Control (*Cxcr4*^*fl/fl*^) and KO (*Cxcr4*^*Ncr1−/−*^) mice. RNA in each group was extracted from NK cells sorted from pooled lymph node and spleen suspensions from 3 mice. **D** Flow cytometry analysis of ILCs in the stroke hemisphere from Control (*Cxcr4*^*fl/fl*^) and KO (*Cxcr4*^*Ncr1−/−*^) mice at P15, NK (CD45^+^Lin^−^NKp46^+^NK1.1^+^CD49a^−^CD49b^+^), intILC1 (CD45^+^Lin^−^NKp46^+^NK1.1^+^CD49a^+^CD49b^+^), ILC1 (CD45^+^Lin^−^NKp46^+^NK1.1^+^CD49a^+^CD49b^−^), ILC2 (CD45^+^Lin^−^NKp46^−^NK1.1^−^CD127^+^ST2^+^KLRG1^+^) and NKp46^+^ ILC3 (CD45^+^Lin^−^NK1.1^−^NKp46^+^CD127^+^). Representative dot plots of ILCs at P15 (*n* = 3 mice) are shown. **E** Quantification of ILCs in the stroke hemisphere from WT (*Cxcr4*^*fl/fl*^) and KO (*Cxcr4*^*Ncr1−/−*^) mice at P0, P2 and P15. Data represent *n* = 3 mice. NK P2 **P = 0.0085, P15 ****P < 0.0001. **F** Analysis at P0 (*n* = 3 mice), 2 (*n* = 4 mice) of the CXCR4^+^ NK cells at the top and total NK cells (Lin^−^CD45^+^NKp46^+^NK1.1^+^CD49b^+^) at the bottom in blood of PT-treated mice showed a significant increase of CXCR4 ^+^ NK cells at P2 and P15 within the blood, while total NK cells were significantly increased only at P15

Of the ILC family, mainly NKp46^+^ (encoded by the *Ncr1* gene) NK cells and ILC1s responded to the stroke lesion (Fig. 2C). Therefore, we used the *Ncr1*^*iCre*^ model to delete *Cxcr4* specifically in these cells. We confirmed *Ncr1*^*iCre*^ validity with the *Ncr1*^*iCre/*+^*; Rosa*^*tdTomato/*+^ reporter model. At P10, 96.4 ± 0.4% of NK cells were tdT^+^ (Fig. 4A). Of the total CD45^+^ population, more than 95% of tdT^+^ cells were lineage negative (Fig. 4B), of which 86.9 ± 2.5% constituted NK cells as well as ILC1s and 7.0 ± 1.5% constituted NKp46^+^ ILC3s(Fig. 4B). Therefore, we concluded that *Ncr1*^*iCre/*+^ mice are a suitable model to drive specific depletion of *Cxcr4* in almost all the NKp46^+^ ILCs. We confirmed the lack of *Cxcr4* expression by sorting the CD45^+^Lin^−^NKp46^+^ cells (Additional file 1: Fig. S5A and S5B) from *Ncr1*^*iCre/*+^*;Cxcr4*^*flox/flox*^, hereafter named *Cxcr4*^*Ncr1−/−*^ mice, and observed drastically reduced *Cxcr4* expression by RT-qPCR (Fig. 4C). Although the fluorescence of cell surface NKp46 was lower in the *Ncr1*^*iCre/*+^ mice, NK and ILC1 cell numbers in the brains and livers of *Ncr1*^*iCre/*+^ mice were comparable with *Cxcr4*^*flox/flox*^ mice (Additional file 1: Figs. S6A and S6B) as reported before in the original description of this line [20]. We induced PT on control (*Cxcr4*^*flox/flox*^ and *Ncr1*^*iCre/*+^) and *Cxcr4*^*Ncr1−/−*^ mice and analyzed the ILC subsets by flow cytometry (Additional file 1: Fig. S4A). We observed that deletion of *Cxcr4* resulted in loss of NK cell migration, while in the control mouse brains NK cell numbers increased at P2 and P15 after PT induction (Fig. 4D, E and Additional file 1: Fig. S6B). At P15 we detected fewer intILC1s in the *Cxcr4*^*Ncr1−/−*^ brain (Fig. 4E). ILC1s, ILC2s and NKp46^+^ ILC3s were not changed in the brain of *Cxcr4*^*Ncr1−/−*^ mice (Fig. 4E). Although the total NK cell number was not significantly increased in the lesion at P2 (Fig. 2B), we observed a significant decrease in NK cell numbers in the *Cxcr4*^*Ncr1−/−*^ lesioned brain at P2 (Fig. 4E). This data suggests that the CXCR4^+^ NK cells migrate preferentially towards the lesion in the first phase after stroke. Indeed, at P2 we noted that CXCR4^+^ NK cells were significantly increased within the blood of mice which received photothrombotic stroke, while the total NK cell population did not change significantly (Fig. 4F). These results validate that CXCL12–CXCR4 axis drives the migration of NK cells into ischemic stroke brain.

### NK cells positively regulate behavioral deficits after PT stroke induction

**Fig. 5:**
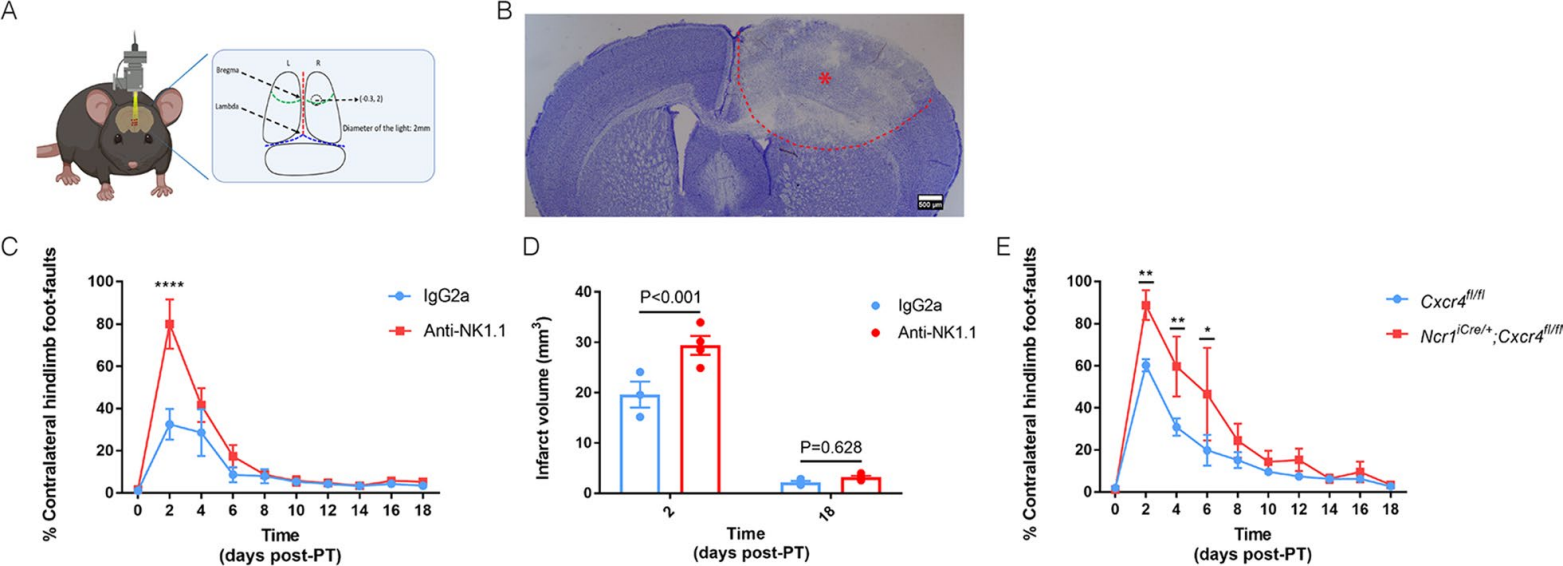
NK cells positively regulate behavioral deficits after PT stroke induction. **A** Coordinates of the lesion on the brain of photothromobotic (PT) mouse model used for beam-walk sensorimotor test analysis. The center of the lesion is 0.3 mm in front of bregma and 2.0 mm lateral to midline, with the 2 mm of diameter. **B** Nissl staining on the vibratome section of stroke brain at day 2 after PT. Data represent *n* = 7 mice. **C** The beam-walk sensorimotor test analysis was performed by calculating percentage of contralateral hindlimb faults. Anti-NK1.1 or IgG2a was injected at day 9 (P-9) and day 2 (P-2) before PT induction and the efficiency of depletion in blood was tested at P-7, P-1, P6, P12 and P18. The videos were recorded just before PT induction (P0) and every second day afterwards. (*n* = 3 mice for IgG2a, *n* = 4 mice for anti-NK1.1). **D** Lesion sizes of brains from the isotype control (IgG2a) (blue) vs. anti-NK1.1 (red) injected mice at P2 and P18 (*n* = 3 mice for IgG2a, *n* = 4 mice for anti-NK1.1). **E** Control (*n* = 4) and KO (*n* = 4) mice after PT induction by calculating the percentage of contralateral hindlimb faults.. P2 **p = 0.0058, P4 **P = 0.0053, P6 **P = 0.0096

Next, we tested whether NK cell presence affected the behavioral outcome of mice after ischemic stroke. To this end, we performed a beam-walk sensorimotor test in mice in which we induced the photothrombotic stroke in the entire cortical area controlling forelimb and hindlimb movement (Fig. 5A, B). We did not observe a significant difference in ILC numbers between this model and the previously used 1-mm-diameter window for ischemic stroke (data not shown). The mice used in this study were trained for 4 consecutive days before PT induction and the analysis of their motor behavior during the beam-walk sensorimotor test was assessed by video and compared to that just before PT induction (day 0) (Additional file 1: Fig. S7A). We recorded the videos every second day and counted the total steps as well as every faulty step or slip in each test.

In order to assess the role of the NK cells, we depleted these cells in C57BL/6J mice by anti-NK1.1 treatment 9 and 2 days before PT induction and confirmed the deletion by flow cytometry before and after PT induction (Additional file 1: Fig. S7A, B). The mice injected with anti-NK1.1 and associated loss of NK cells showed more severe behavioral deficits at P2 (Fig. 5C). Concomitantly, the lesion sizes in the brains with few NK cells were significantly larger at P2 but not at P18, possibly explaining a positive effect of NK cells on the initial motor behavior response recovery (Fig. 5D, Additional file 1: Fig. S7C, D). To specify whether migration via CXCL12/CXCR4 was involved in this process, we specifically and conditionally deleted *Cxcr4* in NK cells, similarly as performed before (Fig. 4D). We observed that loss of NK cells in the *Cxcr4*^*Ncr1−/−*^ mice negatively affected behavioral outcome after PT induction (Fig. 5E). The increased behavioral deficit observed at P2 is consistent with a decreased NK cell number in the brain of P2 *Cxcr4*^*Ncr1−/−*^ mice (Fig. 4E). These findings validate the importance of the CXCL12/CXCR4 axis for the migration of NK cells towards the lesion and recovery after ischemic stroke.

## Discussion

Innate immune cells like neutrophils and macrophages mount an immune reaction after ischemic stroke [4]. However, besides studies on NK cells [7, 27, 34], the presence and role of all other ILCs in ischemic stroke brain was unknown. In this study, we thoroughly analyzed the kinetics of ILCs migration towards the stroke lesion. We have used a minimally invasive and reproducible mouse model using photo-thrombosis (PT), which mainly affected the cortex. In this model, we observed the presence of NK/ILC1s, ILC2s and ILC3s within the brain lesion. Intriguingly, NKp46^+^ ILC3s mainly accumulated in the dorsal region of the infarct area while ILC2s and NKp46^−^ ILC3s are present in the deeper region, suggesting that the mechanisms mediating the migration of ILCs into the lesion are distinct and they play different roles.

ILCs are considered tissue resident [35], although some circulation was reported [32]. We observed very few NK cells and hardly any ILCs in the brain at the moment of stroke induction (P0), or in naïve conditions. Therefore, ILCs observed in the brains were not resident and migrated towards the lesion after stroke induction. We observed that NK cells and ILC1s were the main ILC populations after PT stroke, peaking at P15 and outnumbering the Th17 population. The increase in NK and Th17 cells was consistent with the increase found in the tMCAO model for ischemic stroke [7, 27, 34], confirming our PT model for analyzing ILCs during ischemic stroke in the brain. Even though ILC2s, NKp46^+^ ILC3s and LTi-like cells also increased, they represented a small portion of the infiltrated ILCs. The lack of Ki67 suggests that the robust accumulation of NK cells and ILC1s within the stroke brain was not caused by in situ proliferation, but rather progressive migration towards the ischemic lesion. This is consistent with a previous study in which a correlation between spleen atrophy and presence of NK cells after ischemic stroke in the brain was observed [34], indicating mobilization from the spleen towards the lesion. In the EAE model, it was shown that lymphocytes accumulated within the dura near the lesion [12]. In our PT model, we did not observe an increase of ILCs within the dura during ischemic stroke. Since the meninges did not harbor extra ILCs, there were no ILCs within the brains at steady-state, it is therefore most likely that the ILCs migrated towards the lesion, possibly from the damaged blood vessels within the lesion.

CXCL12 was reported to be involved in the migration of CXCR4^+^ monocytes towards stroke [4, 13]. Moreover, in EAE it was shown that CXCL12 from the BBB was involved in the migration of lymphocytes towards the lesion [36], but the role for CXCL12 from the BBB in the attraction of innate immune cells was not shown before. We deleted CXCL12 in the BBB by using *Cxcl12*^*Cdh5–/–*^ mice, which resulted in decreased NK and NKp46^+^ ILC3 numbers. Even though ILC1s express CXCR4, their migration was not affected in *Cxcl12*^*Cdh5–/–*^ nor in *Cxcr4*^*Ncr1–/–*^ mice. This suggests that their migration to the ischemic lesion occurred through other mechanisms, possibly CX3CL1/CX3CR1 signaling as was shown before for NK cells and intILC1s [7]. The intILC1s were decreased in the ischemic lesions in *Cxcr4*^*Ncr1–/–*^ mice, while not affected in *Cxcl12*^*Cdh5–/–*^ mice. Possibly, intILC1s require a different CXCL12 source than expressed by the BBB, as it is expressed throughout the brain [13]. The lack of NK cells in *Cxcr4*^*Ncr1–/–*^ mice to the lesion in this model suggest that the BBB is essential for the migration of NK cells to the stroke lesion by CXCL12 expression.

The role for Cxcr4^+^ NK cells became clear when we prevented migration of these cells in the *Cxcr4*^*Ncr1–/–*^ model or removing the complete NK cell population via anti-NK1.1-mediated depletion. Using these complementary models, we demonstrated a protective effect of NK cells on recovery of motor deficits in vivo. Other studies have shown positive effects of NK cells in ischemic stroke. Using the *Rag1*^*−/−*^ model, lacking all adaptive lymphocytes but which still had NK cells, showed a lower neurogenic deficiency in ischemic stroke [37]. This data confirms that NK cells in this model could positively affect recovery in this testing. Conversely, in another study it was shown that NK cells had a detrimental effect on stroke [7]. In this study *Rag2*^*−/−*^*γc*^*−/−*^ animals, which constitutively lacked all lymphocytes including NK cells, and anti-NK1.1 treatment was used and tested with the a less sensitive Bederson motor behavior test [38]. Differences between NK cell behavior could be due to different genetic strains or behavioral test. The positive effects of NK cells were shown before in EAE [39, 40], in which NK cells induced homeostatic anti-inflammatory activities of astrocytes via IFN-γ. Indeed, astrocytes were found to be activated in ischemic stroke already at P3 [4], at the same time when the effect of NK cell absence was observed in our beam-walk sensorimotor test. Thus, similar as described in EAE, NK cells could dampen the inflammation via astrocytes in the stroke region to promote recovery of the lesion and motor control. It is of interest to note that the level of NK cell interactions with astrocytes was determined by the level of IFNγ priming of NK cells in the gut, induced by microbiota [39, 40]. Therefore, the microbiota could also play an important role in the recovery after stroke and could also explain the differences between our and other studies [7] in NK cell functionality.

In this study, we thoroughly examined the presence of all ILCs in ischemic stroke. ILCs are not resident within the brain.During ischemic stroke we observed mainly NK cells and, to a lesser extent, intILC1s and ILC1s located at the rim of the lesion. CXCL12 expressed by the BBB attracted the NK cells directly towards the lesion. Deleting this chemokine in endothelial cells, as well as its receptor on the NKp46^+^ cells, prevented migration towards the lesion. CXCR4 is important for the attraction of the NK cells, and other innate immune cells towards stroke, indirectly affecting microglial function [4] and was shown in both studies to be positively associated with stroke recovery. NK cells have been detected in ischemic lesions in humans [7, 34], and it was suggested to restrict NK cell migration towards the lesion to prevent secondary pneumonia using, e.g., AMD3100 [34]. However, our data indicate that the migration of CXCR4^+^ NK cells could be beneficial for recovery. The positive effects of NK cells were shown in EAE before [41]. Similarly, CXCR4^+^ NK cells were observed specifically during the remission phase in multiple sclerosis [18]. If the role of NK cells and other ILC1 members is similar in ischemic lesions of patients remains to be established. The deeper penetration of ILC2s and NKp46^−^ILC3s suggests that the mechanisms underlying the migration of ILCs into stoke lesion are distinct.

## Supplementary Information

**Additional file 1: Figure S1.** Gating strategy for flow cytometry analysis on *RORc*^*eGFP*^-reporter mice. Gating strategy for FACS on brain hemisphere with the lesion (A), dura mater (B) and lymph node (C) samples, NK (CD45^+^Lin^−^NKp46^+^CD127^−^), ILC1 (CD45^+^Lin^−^NKp46^+^CD127^+^RORγt^−^), ILC2 (CD45^+^Lin^−^NKp46^−^CD127^+^RORγt^−^ST2^+^KLRG1^+^), NKp46^+^ ILC3 (CD45^+^Lin^−^NKp46^+^CD127^+^RORγt^+^), LTi-like (CD45^+^Lin^−^NKp46^−^CD127^+^RORγt^+^), and Th17 (CD45^+^Lin^+^CD4^+^RORγt^+^). Representative dot plots of ILC and Th17 cells at day 15 (*n* = 3 mice) are shown. **Figure S2.** Quantification of ILCs and Th17 cells in the dura mater and lymph nodes after PT induction. FACS analysis of ILCs in the dura mater (A), NK (CD45^+^Lin^−^NKp46^+^CD127^−^), ILC1 (CD45^+^Lin^−^NKp46^+^CD127^+^RORγt^−^), ILC2 (CD45^+^Lin^−^NKp46^−^CD127^+^RORγt^−^ST2^+^KLRG1^+^), NKp46^+^ ILC3 (CD45^+^Lin^−^NKp46^+^CD127^+^RORγt^+^), LTi-like (CD45^+^Lin^−^NKp46^−^CD127^+^RORγt^+^), and Th17 (CD45^+^Lin^+^CD4^+^RORγt^+^). Representative dot plots of ILCs and Th17 cells at P15 (*n* = 3 mice, 3 independent experiments) are shown. (B) Quantification of ILC populations and Th17 cells in the dura mater at different time points after PT induction. N = 3 mice for each group at each time point, 3 independent experiments.. (C) Flow cytometry analysis of ILCs in the lymph nodes, representative dot plots of ILCs and Th17 cells at day 15 are shown. Quantification of ILC and Th17 cells in the lymph nodes including (D) deep cervical lymph node (dcLN, *n* = 3 mice for each group at each time point except for *n* = 2 mice for PT at P4), (E) mandibular LN (mandiLN, *n* = 3 mice for each group at P0, P2, P4, P10 and P15; *n* = 2 mice for each group at P20), (F) mesenteric LN (mLN, *n* = 3 mice for each group at P0, P2, P4, P10 and P15; *n* = 2 mice for each group at P20) and (G) axillary LN (axiLN, *n* = 3 mice for each group at P0, P2, P4, P10 and P15; *n* = 2 mice for each group at P20). **Figure S3.** Sorting endothelial cells. Gating strategy for sorting endothelial cells (CD31^+^CD45^−^) from WT (*Cxcl12*^*fl/fl*^, *n* = 3) (A) and KO (*Cxcl12*^*Cdh5–/–*^, *n* = 3) (B) mice. **Figure S4.** Gating strategy for flow cytometry on *Cdh5*^*CreERT2*^*;Cxcl12*^*fl*^ and *Ncr1*^*iCre*^*;Cxcr4*^*fl*^ mice. Gating strategy for FACS on brain hemisphere with the lesion (A), dura mater (B) and lymph node (C) samples; NK (CD45^+^Lin^−^NKp46^+^NK1.1^+^CD49a^−^CD49b^+^), intILC1 (CD45^+^Lin^−^NKp46^+^NK1.1^+^CD49a^+^CD49b^+^), ILC1 (CD45^+^Lin^−^NKp46^+^NK1.1^+^CD49a^+^CD49b^−^), ILC2 (CD45^+^Lin^−^NKp46^−^NK1.1^−^CD127^+^ST2^+^KLRG1^+^) and NKp46^+^ ILC3 (CD45^+^Lin^−^NK1.1^−^NKp46^+^CD127^+^). Representative dot plots of ILCs at day 15 (*n* = 3) are shown. **Figure S5.** Sorting NKp46^+^ ILC cells. Gating strategy for sorting NKp46^+^ ILC cells (CD45^+^Lin^−^NKp46^+^) from WT (*Cxcr4*^*fl/fl*^, *n* = 3)) (A) and KO (*Cxcr4*^*Ncr1−/−*^, *n* = 3) (B) mice. Lin: CD3e, CD8a, CD19, Ly6G, TCRβ, F4/80. **Figure S6.** Analysis of NK1.1^+^NKp46^+^ ILC cell numbers in the *Cxcr4*^*fl/fl*^ and *Ncr*^*iCre/*+^ mice. (A) FACS analysis of NK and ILC1 cells in the liver of *Cxcr4*^*fl/fl*^ and *Ncr*^*iCre/*+^ mice. (B) Analysis of NK cells in the PT stroke brain of *Cxcr4*^*fl/fl*^ (*n* = 3) and *Ncr*^*iCre/*+^ (*n* = 3) mice, at P0, P2 and P15. **Figure S7.** Strategy for the anti-NK1.1-mediated depletion of NK cells. (A) Anti-NK1.1 or IgG2a was injected at day 9 (P-9) and day 2 (P-2) before PT induction and the efficiency of depletion in blood was tested at P-7, P-1, P6, P12 and P18. The videos were recorded just before PT induction (P0) and every second day afterwards. (B) Gating strategy for analyzing the presence of NK cells in the blood. Lin: CD3e, CD8a, CD19, Ly6G, TCRβ, F4/80. NK cell depletion was established at P0, showed in a representative flow cytometry analysis of the brains from a control (isotype IgG2a, top) and the anti-NK1.1 treated mice (bottom) (*n* = 2 for each time point per treatment). (C) Nissl staining of the lesion in representative series of a mouse brain at P2 after stroke and (D) P18 after stroke, indicating decrease of lesion size. Control (IgG2a) brains are on the left, while the anti-NK1.1 depleted brains are shown on the right (*n* = 3 for IgG2a, *n* = 4 for anti-NK1.1).

**Additional file 2: Video S1.** 3D imaging of the brain lesion from a stroke mouse. Whole mount staining for the stroke brain at day 2 after PT induction. In the original acquisition KLRG1 in shown in cyan, RORγt in green, CD3 in red, NKp46 in white. Using the spot function in Imaris, we determined NK cells, ILC1s and NKp46^+^ ILC3s (CD3^–^NKp46^+^) as white spots, ILC3s and LTi cells (CD3^–^RORγt^+^) as green spots and ILC2s (CD3^–^KLRG1^+^) as cyan spots. Data represent *n* = 2 mice.

**Additional file 3: Video S2.** 3D imaging of the brain lesion from a stroke mouse. Whole mount staining for the stroke brain at day 10 after PT induction. In the original acquisition KLRG1 in shown in cyan, RORγt in green, CD3 in red, NKp46 in white. Using the spot function in Imaris, we determined NK cells, ILC1s and NKp46^+^ ILC3s (CD3^–^NKp46^+^) as white spots, ILC3s and LTi cells (CD3^–^RORγt^+^) as green spots and ILC2s (CD3^–^KLRG1^+^) as cyan spots. Data represent *n* = 2 mice.

## Data Availability

Further information and requests for resources and reagents should be directed to and will be fulfilled by the corresponding author (vandepavert@ciml.univ-mrs.fr). This study did not generate new unique reagents.
