# Supplementary material for: Brain endothelial CXCL12 attracts protective natural killer cells during ischemic stroke
Source: J Neuroinflammation. 2023 Jan 11;20:8. doi: 10.1186/s12974-023-02689-x (PMC9835334; doi:10.1186/s12974-023-02689-x)
Supplement: Supplementary file 1 — Additional file 1: Figure S1. Gating strategy for flow cytometry analysis on RORceGFP-reporter mice. Gating strategy for FACS on brain hemisphere with the lesion (A), dura mater (B) and lymph node (C) samples, NK (CD45+Lin−NKp46+CD127−), ILC1 (CD45+Lin−NKp46+CD127+RORγt−), ILC2 (CD45+Lin−NKp46−CD127+RORγt−ST2+KLRG1+), NKp46+ ILC3 (CD45+Lin−NKp46+CD127+RORγt+), LTi-like (CD45+Lin−NKp46−CD127+RORγt+), and Th17 (CD45+Lin+CD4+RORγt+). Representative dot plots of ILC and Th17 cells at day 15 (n = 3 mice) are shown. Figure S2. Quantification of ILCs and Th17 cells in the dura mater and lymph nodes after PT induction. FACS analysis of ILCs in the dura mater (A), NK (CD45+Lin−NKp46+CD127−), ILC1 (CD45+Lin−NKp46+CD127+RORγt−), ILC2 (CD45+Lin−NKp46−CD127+RORγt−ST2+KLRG1+), NKp46+ ILC3 (CD45+Lin−NKp46+CD127+RORγt+), LTi-like (CD45+Lin−NKp46−CD127+RORγt+), and Th17 (CD45+Lin+CD4+RORγt+). Representative dot plots of ILCs and Th17 cells at P15 (n = 3 mice, 3 independent experiments) are shown. (B) Quantification of ILC populations and Th17 cells in the dura mater at different time points after PT induction. N = 3 mice for each group at each time point, 3 independent experiments.. (C) Flow cytometry analysis of ILCs in the lymph nodes, representative dot plots of ILCs and Th17 cells at day 15 are shown. Quantification of ILC and Th17 cells in the lymph nodes including (D) deep cervical lymph node (dcLN, n = 3 mice for each group at each time point except for n = 2 mice for PT at P4), (E) mandibular LN (mandiLN, n = 3 mice for each group at P0, P2, P4, P10 and P15; n = 2 mice for each group at P20), (F) mesenteric LN (mLN, n = 3 mice for each group at P0, P2, P4, P10 and P15; n = 2 mice for each group at P20) and (G) axillary LN (axiLN, n = 3 mice for each group at P0, P2, P4, P10 and P15; n = 2 mice for each group at P20). Figure S3. Sorting endothelial cells. Gating strategy for sorting endothelial cells (CD31+CD45−) from WT (Cxcl12fl/fl, n = 3) (A) and KO (Cxcl12Cdh5–/–, n = 3) [file 12974_2023_2689_MOESM1_ESM.pdf]

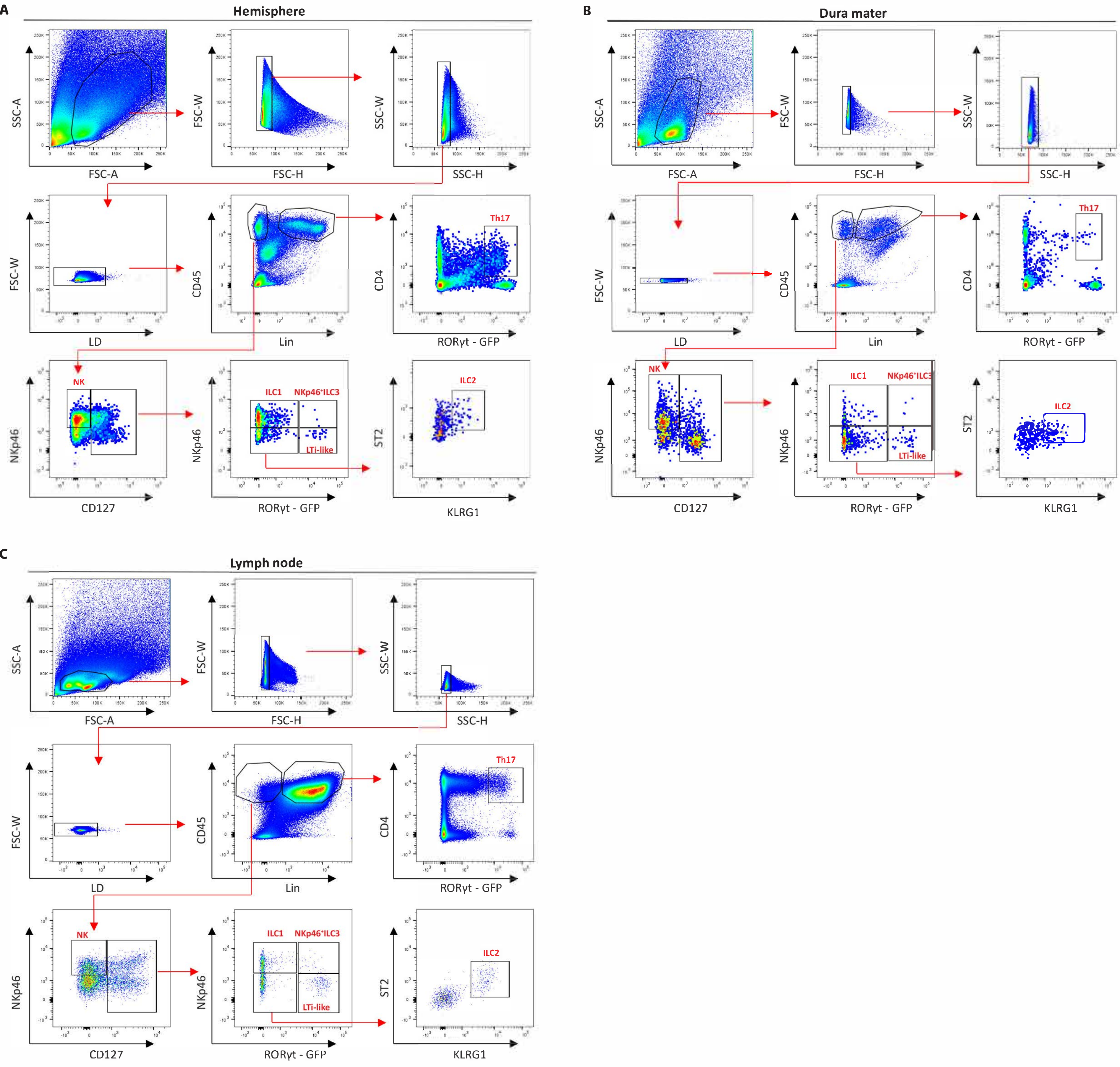

Fig. S1: Gating strategy for flow cytometry analysis on *RORc^{eGFP}*- reporter mice. Gating strategy for FACS on brain hemisphere with the lesion (A), dura mater (B) and lymph node (C) samples, NK (CD45$^+$Lin$^-$NKp46$^+$CD127$^-$), ILC1 (CD45$^+$Lin$^-$NKp46$^+$CD127$^+$RORγt$^-$), ILC2 (CD45$^+$Lin$^-$NKp46$^-$CD127$^+$RORγt$^-$ST2$^+$KLRG1$^+$), NKp46$^+$ ILC3 (CD45$^+$Lin$^-$NKp46$^+$CD127$^+$RORγt$^+$), LTi-like (CD45$^+$Lin$^-$NKp46$^-$CD127$^+$RORγt$^+$), and Th17 (CD45$^+$Lin$^+$CD4$^+$RORγt$^+$). Representative dot plots of ILC and Th17 cells at day 15 (n=3 mice) are shown.

Supplementary Figure 2

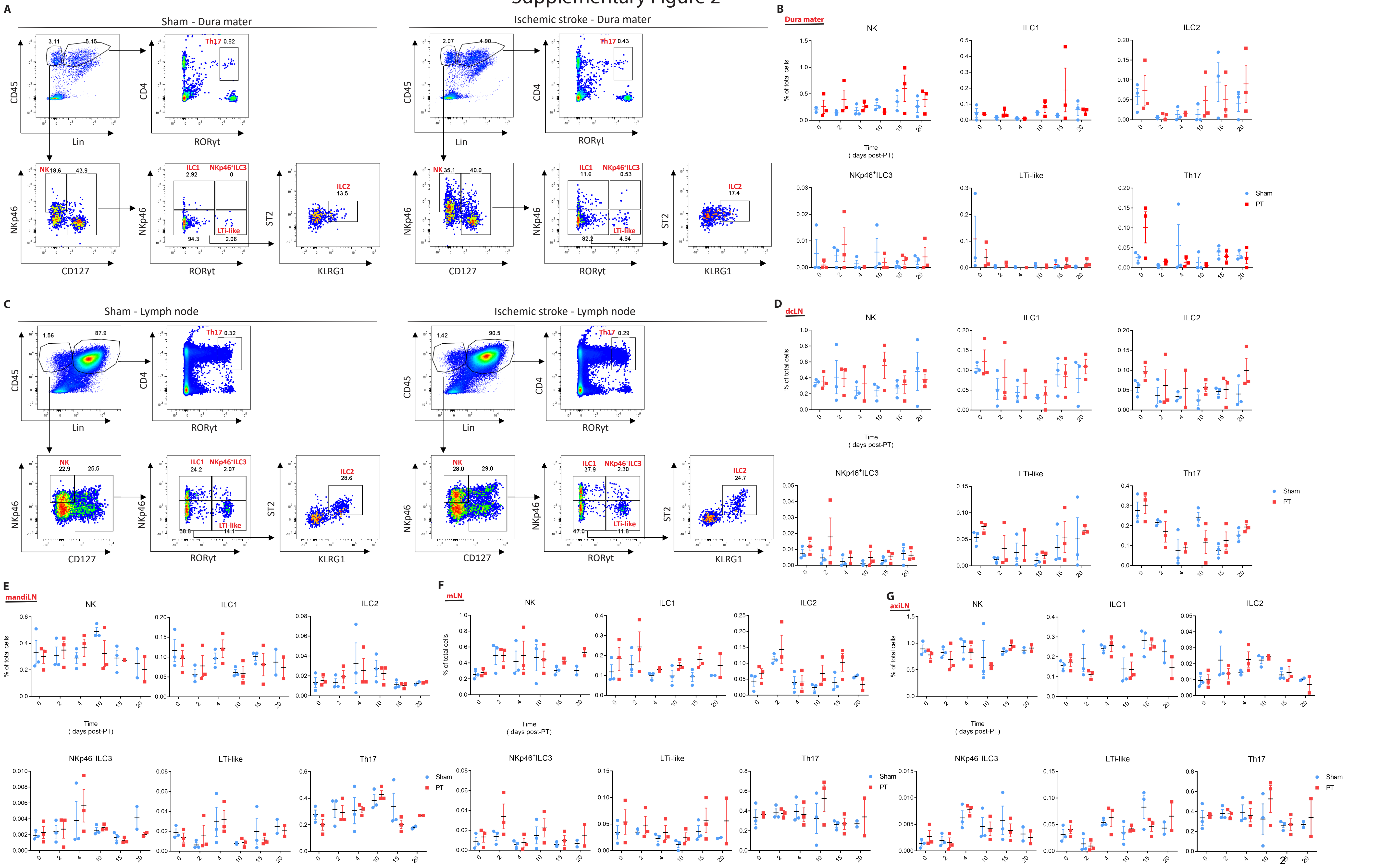

Fig. S2: Quantification of ILCs and Th17 cells in the dura mater and lymph nodes after PT induction. FACS analysis of ILCs in the dura mater (A), NK ($CD45^+Lin^-NKp46^+CD127^-$), ILC1 ($CD45^+Lin^-NKp46^+CD127^+ROR\gamma t^-$), ILC2 ($CD45^+Lin^-NKp46^-CD127^+ROR\gamma t^-ST2^+KLRG1^+$), $NKp46^+$ ILC3 ($CD45^+Lin^-NKp46^+CD127^+ROR\gamma t^+$), LTi-like ($CD45^+Lin^-NKp46^-CD127^+ROR\gamma t^+$), and Th17 ($CD45^+Lin^+CD4^+ROR\gamma t^+$). Representative dot plots of ILCs and Th17 cells at P15 (n=3 mice, 3 independent experiments) are shown. (B) Quantification of ILC populations and Th17 cells in the dura mater at different time points after PT induction. N=3 mice for each group at each time point, 3 independent experiments. (C) Flow cytometry analysis of ILCs in the lymph nodes, representative dot plots of ILCs and Th17 cells at day 15 are shown. Quantification of ILC and Th17 cells in the lymph nodes including (D) deep cervical lymph node (dcLN, n=3 mice for each group at each time point except for n=2 mice for PT at P4), (E) mandibular LN (mandiLN, n=3 mice for each group at P0, P2, P4, P10 and P15; n=2 mice for each group at P20), (F) mesenteric LN (mLN, n=3 mice for each group at P0, P2, P4, P10 and P15; n=2 mice for each group at P20) and (G) axillary LN (axiLN, n=3 mice for each group at P0, P2, P4, P10 and P15; n=2 mice for each group at P20).

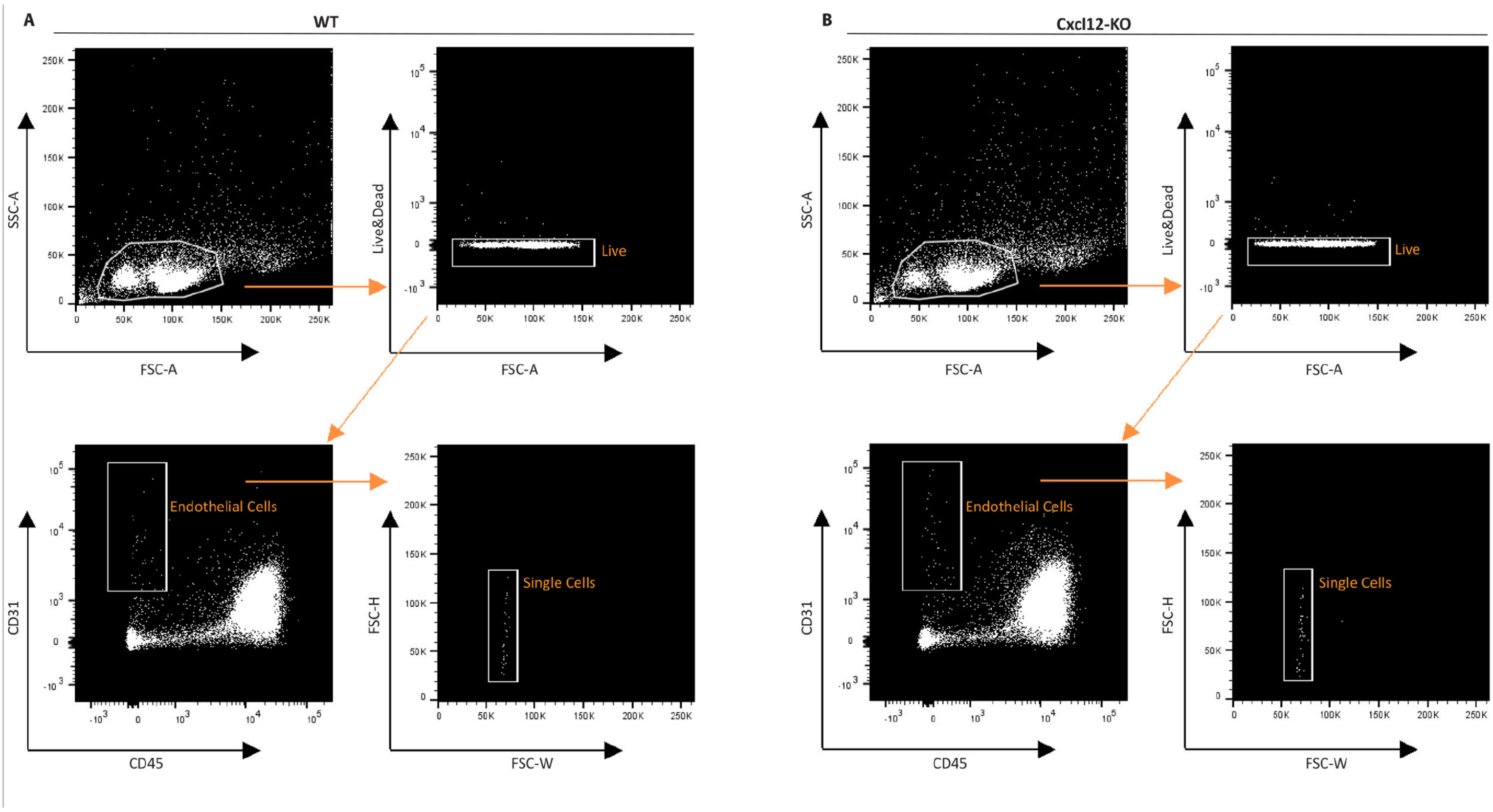

Fig. S3: Sorting endothelial cells. Gating strategy for sorting endothelial cells ($CD31^+CD45^-$) from WT (*Cxcl12*$^{fl/fl}$, n=3) (A) and KO (*Cxcl12*$^{Cdh5-/-}$, n=3) (B) mice.

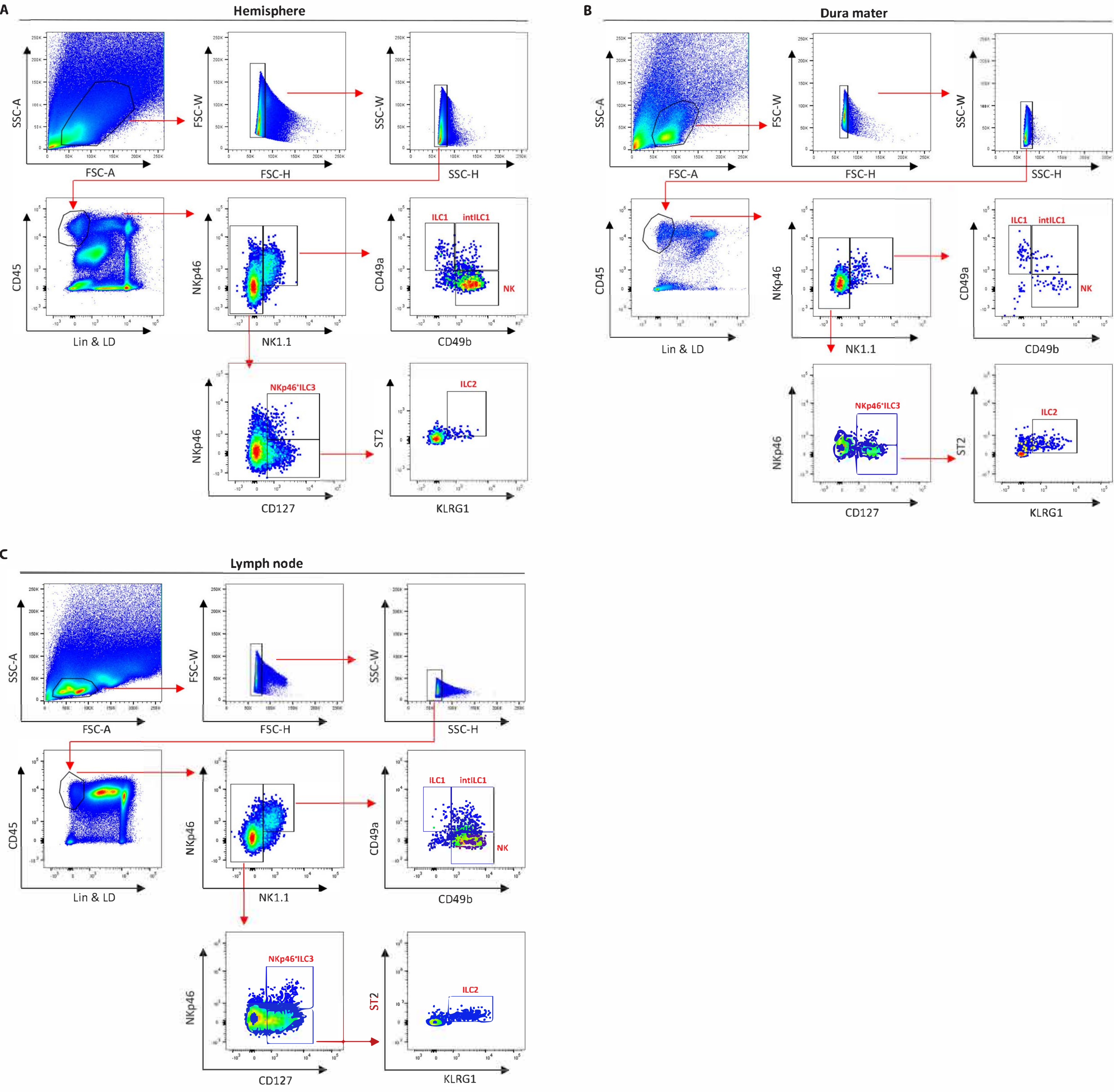

Fig. S4: Gating strategy for flow cytometry on *Cdh5^{CreERT2};Cxcl12^{fl}* and *Ncr1^{iCre};Cxcr4^{fl}* mice. Gating strategy for FACS on brain hemisphere with the lesion (A), dura mater (B) and lymph node (C) samples; NK ($CD45^+Lin^-NKp46^+NK1.1^+CD49a^-CD49b^+$), intILC1 ($CD45^+Lin^-NKp46^+NK1.1^+CD49a^+CD49b^+$), ILC1 ($CD45^+Lin^-NKp46^+NK1.1^+CD49a^+CD49b^-$), ILC2 ($CD45^+Lin^-NKp46^-NK1.1^-CD127^+ST2^+KLRG1^+$) and $NKp46^+$ ILC3 ($CD45^+Lin^-NK1.1^-NKp46^+CD127^+$). Representative dot plots of ILCs at day 15 (n=3) are shown.

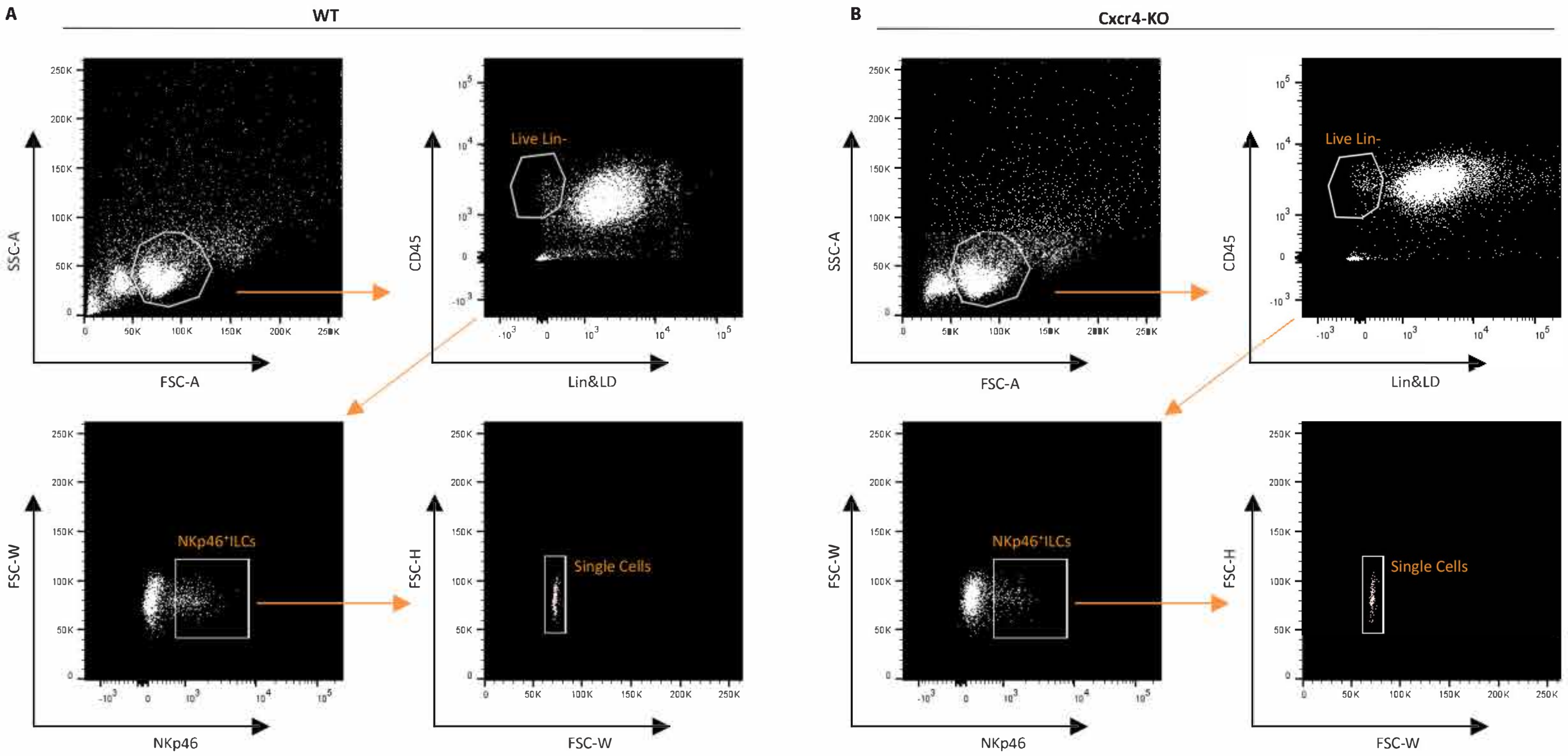

Fig. S5: Sorting NKp46⁺ ILC cells. Gating strategy for sorting NKp46⁺ ILC cells (CD45⁺Lin⁻NKp46⁺) from WT (*Cxcr4*$^{fl/fl}$, n=3)) (A) and KO (*Cxcr4*$^{Ncr1-/-}$, n=3) (B) mice. Lin: CD3e, CD8a, CD19, Ly6G, TCRβ, F4/80.

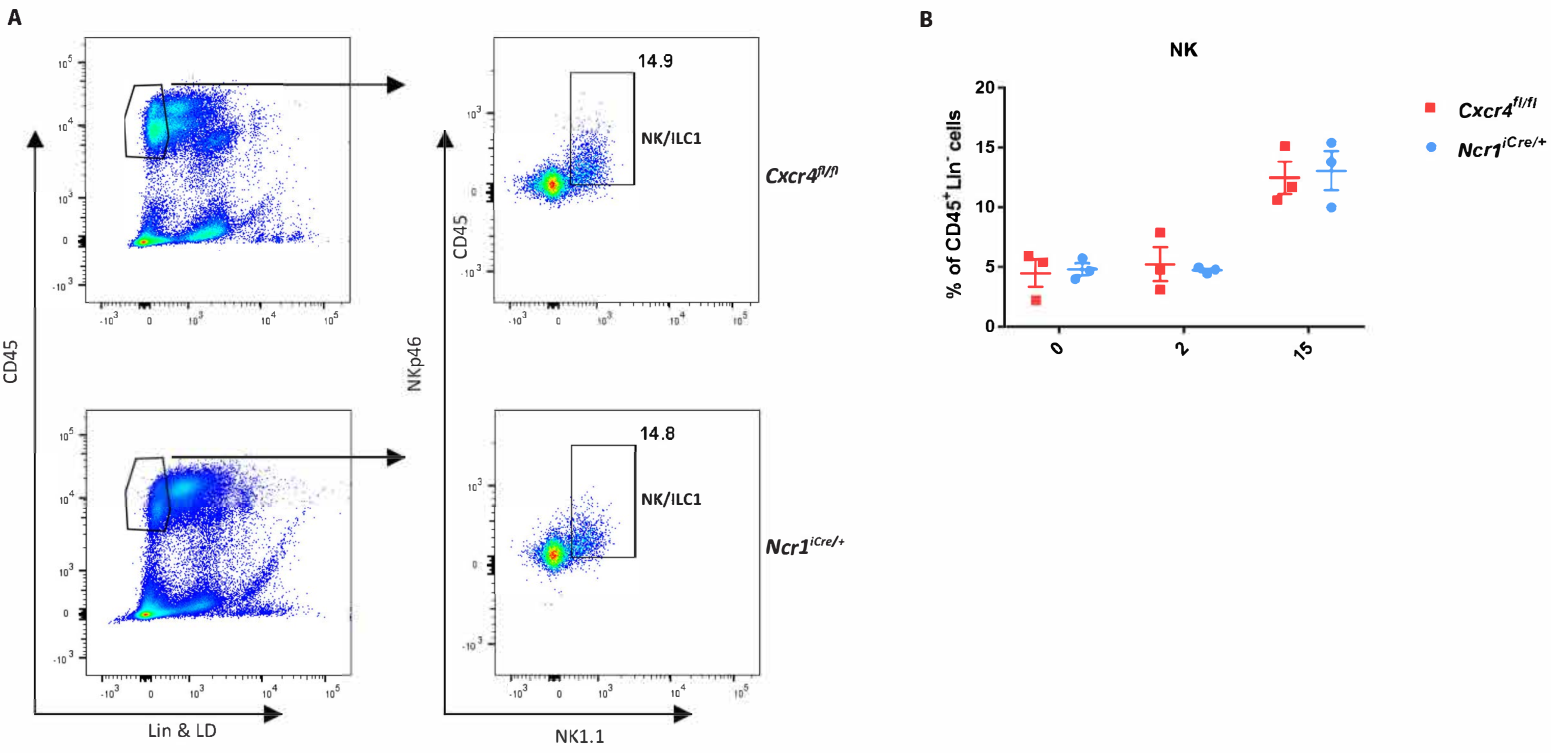

Fig. S6: Analysis of NK1.1⁺NKp46⁺ ILC cell numbers in the *Cxcr4*$^{fl/fl}$ and *Ncr*$^{iCre/+}$ mice. (A) FACS analysis of NK and ILC1 cells in the liver of *Cxcr4*$^{fl/fl}$ and *Ncr*$^{iCre/+}$ mice. (B) Analysis of NK cells in the PT stroke brain of *Cxcr4*$^{fl/fl}$ (n=3) and *Ncr*$^{iCre/+}$ (n=3) mice, at P0, P2 and P15.

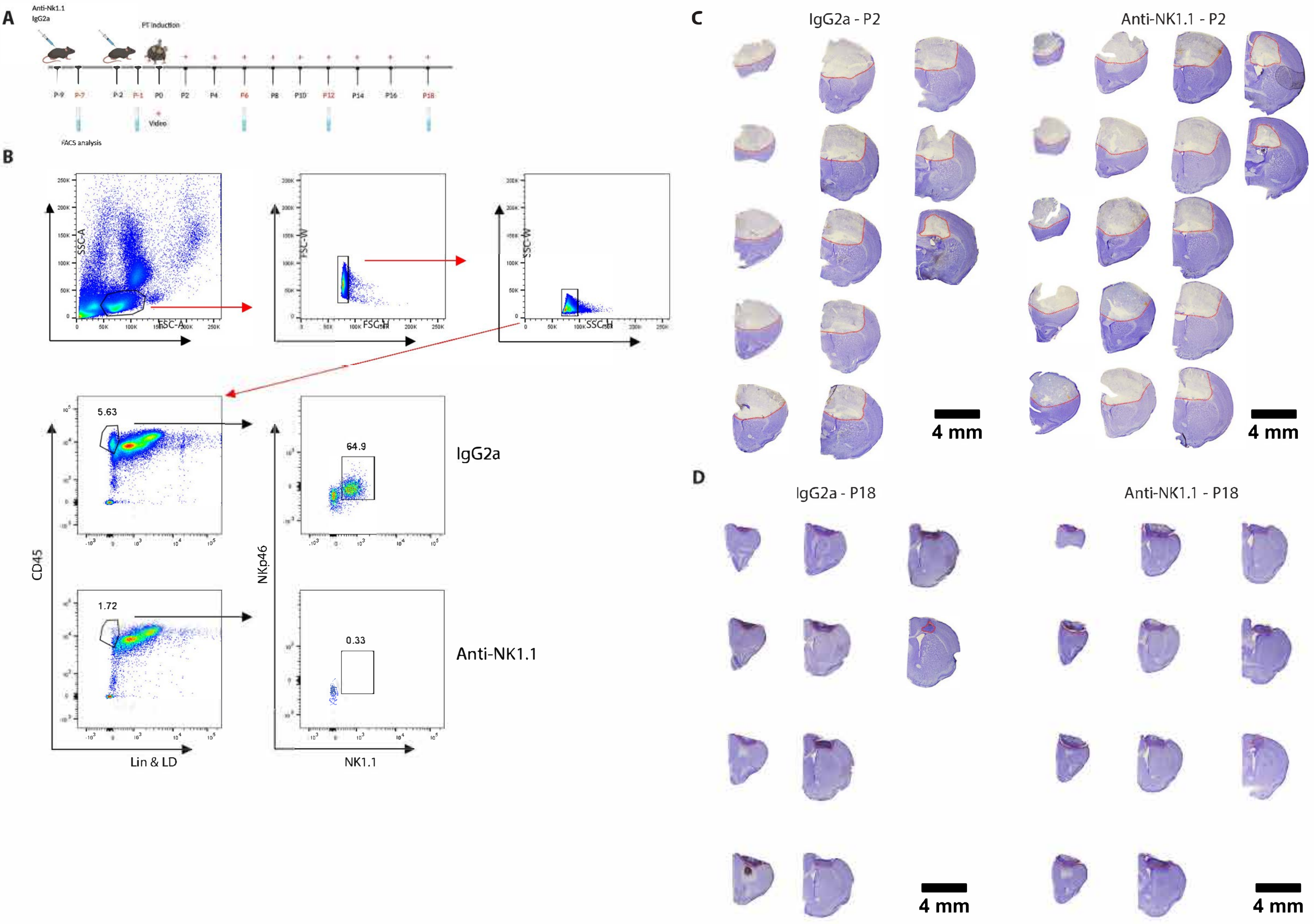

Fig. S7: Strategy for the anti-NK1.1 mediated depletion of NK cells. (A) Anti-NK1.1 or IgG2a was injected at day 9 (P-9) and day 2 (P-2) before PT induction and the efficiency of depletion in blood was tested at P-7, P-1, P6, P12 and P18. The videos were recorded just before PT induction (P0) and every second day afterwards. (B) Gating strategy for analyzing the presence of NK cells in the blood. Lin: CD3e, CD8a, CD19, Ly6G, TCRβ, F4/80. NK cell depletion was established at P0, showed in a representative flow cytometry analysis of the brains from a control (isotype IgG2a, top) and the anti-NK1.1 treated mice (bottom) (n=2 for each time point per treatment). (C) Nissl staining of the lesion in representative series of a mouse brain at P2 after stroke and (D) P18 after stroke, indicating decrease of lesion size. Control (IgG2a) brains are on the left, while the anti-NK1.1 depleted brains are shown on the right (n=3 for IgG2a, n=4 for anti-NK1.1).
